# Insights from the molecular docking analysis of EGFR antagonists

**DOI:** 10.6026/97320630019260

**Published:** 2023-03-31

**Authors:** Mohammad Azhar Kamal, Hanadi M Baeissa, Israa J. Hakeem, Reem S Alazragi, Mohannad S.Hazzazi, Tahani Bakhsh, Akhmed Aslam, Bassem Refaat, Elshiekh B. Khidir, Kadhem Juma Alkhenaizi, Qamre Alam

**Affiliations:** 1 Department of Pharmaceutics, College of Pharmacy, Prince Sattam Bin Abdulaziz University, Alkharj 11942, Saudi Arabia; 2 Department of Biochemistry, College of Science, University of Jeddah, Jeddah, Saudi Arabia; 3 Department of Medical Laboratory Sciences, Faculty of Applied Medical Sciences, King Abdulaziz University, Jeddah 22254, Saudi Arabia; 4 Hematology Research Unit, King Fahad Medical Research Center, King Abdulaziz University, Jeddah 22254, Saudi Arabia; 5 Department of Biology, College of Science, University of Jeddah, Jeddah, Saudi Arabia, P.O. Box 80327, Jeddah 21589; 6 Laboratory Medicine Department, Faculty of Applied Medical Sciences, Umm Al-Qura University, Makkah, Saudi Arabia; 7 Molecular Genomics and Precision Medicine, ExpressMed Laboratories, Block, 359, Zinj,Kingdom of Bahrain

**Keywords:** Cancer, *Glycyrrhiza glabra*, bioactive compounds, cancer, EGFR

## Abstract

Overexpression of the epidermal growth factor receptor (EGFR) has been shown to be a critical factor in tumor development and cancer progression. Although established EGFR inhibitors have been effective in the treatment of cancer, they are associated with several side effects. As a result, there is an urgent need to develop novel EGFR inhibitors that can effectively target the receptor while causing no adverse side effects. Here, the bioactive compounds of *Glycyrrhiza glabra* and established EGFR inhibitors have been screened against the EGFR catalytic site. The compounds LTS0058805, LTS0114552, LTS0128805, LTS0174203, LTS0007447, and LTS0164690 exhibited binding energies to the EGFR that were comparable to those of established EGFR inhibitors. Further, these hit compounds were observed to interact with critical residues of the EGFR, suggesting their potential as inhibitors of the receptor. In addition, these hits possess good drug-like properties and merit further exploration for their potential application in cancer management.

## Background:

**Figure 1:**
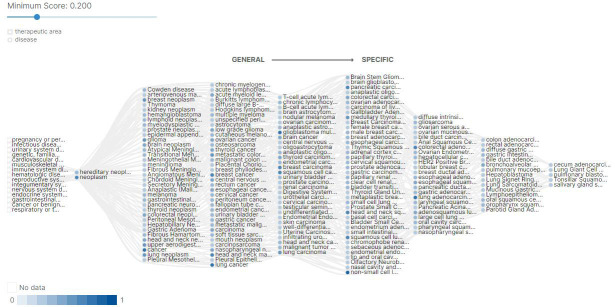
Diseases or phenotypes associated with EGFR predicted by Open Targets Platform.

The epidermal growth factor receptor (EGFR) is located on the surface of epithelial cells and pertains to the family of tyrosine kinase receptors. It is activated by various endogenous ligands, including epidermal growth factor (EGF), which initiates signaling pathways to resume stable cellular functions [1]. Under normal circumstances, EGFR is essential for the development and maintenance of tissues [2]. However, overexpression of EGFR can promote the growth and progression of tumors, resulting in angiogenesis, invasion, and metastasis via multiple pathways, including Ras/Raf/MAPK, PIK-3/AKT, PLC-PKC, and STAT [3]. It is frequently overexpressed in a variety of cancers, including non-small cell lung cancer, cancer of the head and neck, pancreatic cancer, and certain colorectal and breast cancers [4]. Due to the limitations of conventional chemotherapy, such as high toxicity and low tumor sensitivity, EGFR has emerged as a crucial therapeutic target for cancer treatment [5]. EGFR has been linked to a variety of cancers, and its mutations, primarily missense mutations, inframe deletions, and others, have also been documented [6,7]. There are 764 'cancer or benign tumor' diseases or phenotypes associated with EGFR, predicted by the 'Open Targets Platform' [8]. The Open Targets Platform is a powerful resource that facilitates the methodical discovery and ranking of promising targets for therapeutic drugs. Several inhibitors are currently approved for use in cancer treatment, such as Erlotinib (Tarceva), Gefitinib (Iressa), Afatinib (Gilotrif), Osimertinib (Tagrisso), Lapatinib (Tykerb), Cetuximab (Erbitux), Panitumumab (Vectibix), Necitumumab (Portrazza), Dacomitinib (Vizimpro), Poziotinib (NOV120101), and many are in clinical trials. Dermatologic adverse effects of EGFR inhibitors include papulopustular eruptions, xerosis and pruritus, nail and hair changes, mucositis, itching, and dryness [9,10]. Therefore, there is a need to design novel inhibitors with no side effects. Natural products have played an important role in the development of anticancer drugs. Many anticancer agents that are commonly used in clinical practice are derived from natural sources [11]. Several plant-derived compounds, such as irinotecan, vincristine, etoposide, and paclitaxel, as well as bacterial-derived compounds such as actinomycin D and mitomycin C, and a marine-derived compound known as bleomycin, have been identified as potential cancer therapeutic agents [12]. Some of these agents are still used in cancer therapy and will play an important role in the near future. Camptothecin and taxol are likely the two most effective examples; both were discovered between the 1950s and 1960s as part of a program launched by the National Cancer Institute to find the therapeutic potential of natural products [12,13]. The goal of this study was to find new candidate molecules from the bioactive compounds of *Glycyrrhiza glabra* (G. glabra) that could potentially serve as EGFR inhibitors in cancer treatment(Figure 1).

## Methodology:

## Retrieval and preparation of known EGFR inhibitors and active compounds of G. glabra:

Using the ChEMBL database (https://www.ebi.ac.uk/chembl/), 15 known EGFR inhibitors were obtained in "sdf" format. Then, Discovery Studio (DS) was used to minimize and convert them to pdbqt file types. ChEMBL is a database of biologically active molecules with drug-like properties that are manually curated. The active compounds of G. glabra were extracted from the LOTUS database [14], one of the most comprehensive and well-annotated natural compound databases, and prepared for virtual screening using DS.

## Retrieval and preparation of the 3D structure of EGFR:

The 3D structure of the EGFR was obtained from the Protein Data Bank. Several crystal structures of EGFR have been reported that capture distinct snapshots of the protein's conformation and are co-crystallized with various inhibitors. This research utilized 1M17, which is a co-crystallized EGFR tyrosine kinase domain with the 4-anilinoquinazoline inhibitor erlotinib [15].

## Virtual screening:

The PyRx program's built-in AutoDock VINA was utilized to conduct virtual screening (VS) of active compounds from G. glabra and known inhibitors of EGFR, against the active site of a prepared target protein, EGFR [16]. The selection of the grid was based on the specific site of the molecule, which was utilized for docking-based VS. The grid box center was established at X = 23.24, Y = -0.4519., and Z = 56.12.

## Physicochemical and druglikeness property prediction:

The pharmacokinetics, efficacy, and safety profiles of these selected hits were predicted using the DataWarrior tool [17].

## Results and Discussion:

**Figure 2:**
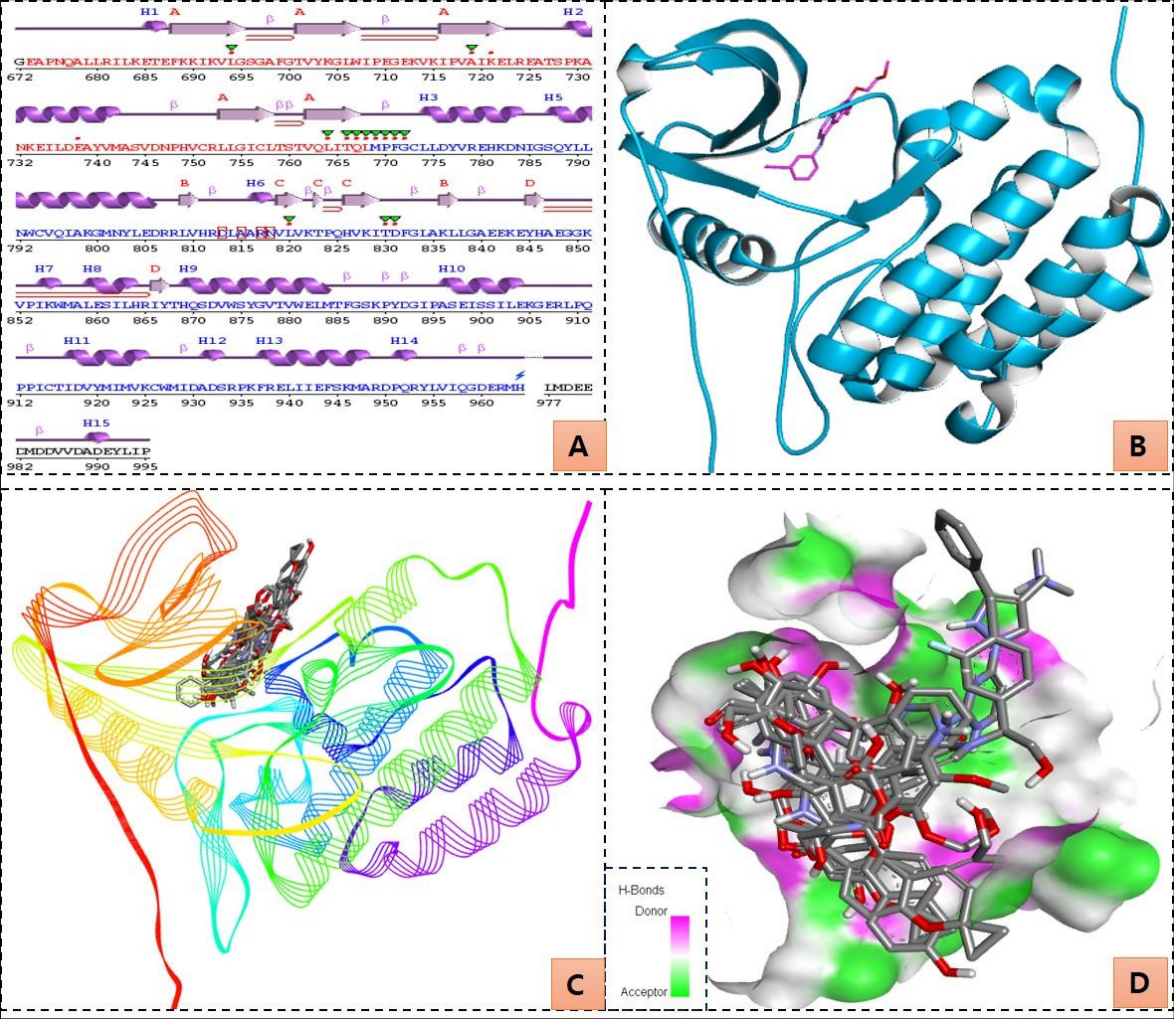
EGFR protein structure and computational screening of compounds targeting its active site. 2D structure (A), 3D structure (B), docked compounds in the active pocket of EGFR (C), and closed view of docked compounds in the active pocket of EGFR (D).

EGFR overexpression has been shown to play a critical role in tumorigenesis and cancer progression [4]; thus, targeting EGFR represents a promising strategy for cancer management. Here, the bioactive compounds of G. glabra and established EGFR inhibitors have been screened against the EGFR catalytic site. The EGFR structure has been extensively studied, and several well-known inhibitors have a robust binding affinity for it. We have illustrated the 2D and 3D structures of EGFR, as well as the binding poses of inhibitors and compounds to the active site residues of G. glabra (Figure 2 A-D). Notably, some of the bioactive compounds of G. glabra display binding energies (BEs) towards EGFR that are similar to those of established EGFR inhibitors (Table 1 and Table 2>). A low (highly negative) BE value indicates greater stability of the ligand with the target protein [18]. Interestingly, compounds LTS0058805, LTS0114552, LTS0128805, LTS0174203, LTS0007447, and LTS0164690 exhibit comparable BEs for EGFR when compared to the control compounds, implying that these aforementioned compounds may possess similar or potentially enhanced therapeutic efficacy as compared to the control compounds. Furthermore, while some compounds demonstrated strong binding affinity towards EGFR, they were found to possess inadequate drug-likeness properties. Table 3 depicts the physicochemical and drug-likeness properties of the best 20 hits.

**Figure 3:**
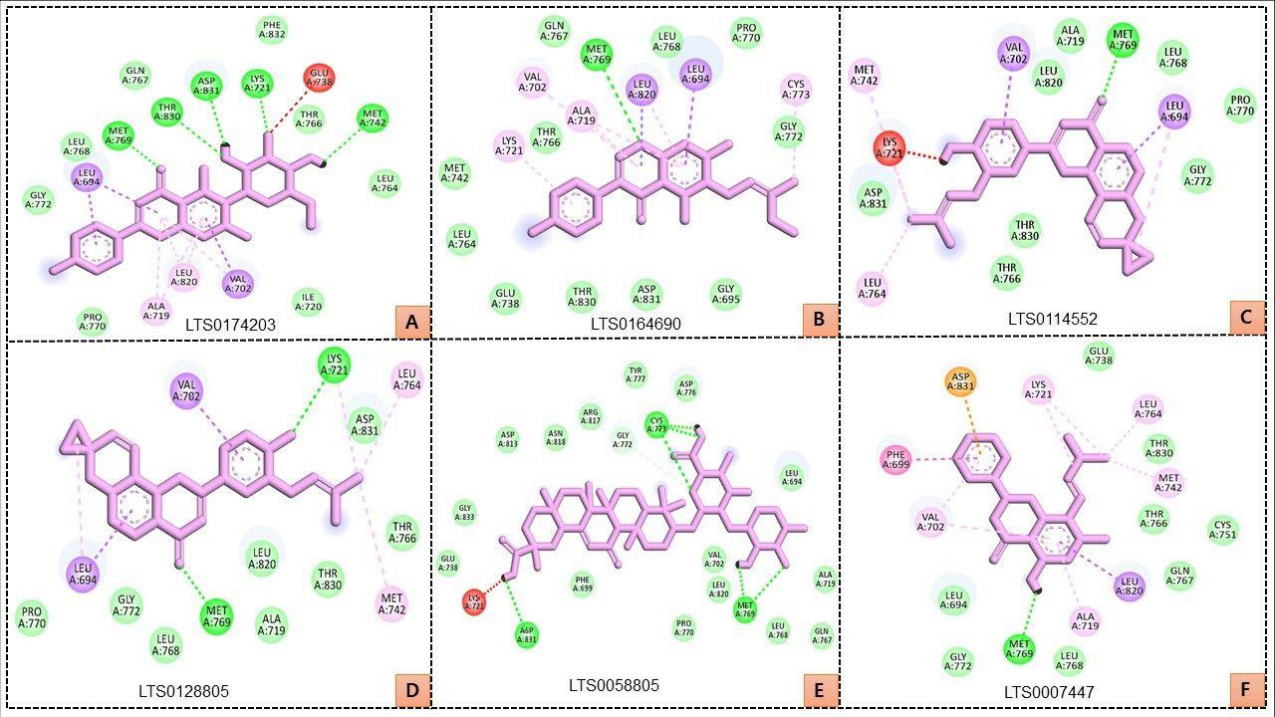
Interacting residues of EGFR with LTS0174203 (A), LTS0164690 (B), LTS0114552 (C), LTS0128805 (D), LTS0058805 (E), and LTS0007447 (F)

The compounds LTS0058805, LTS0114552, LTS0128805, LTS0174203, LTS0007447, and LTS0164690 were selected for in-depth interaction analysis. LTS0174203 interacted with Glu738, Thr766, Met742, Leu764, Ile720, Val702, Leu820, Ala719, Pro770, Gly772, Leu694, Leu768, Met769, Thr830, Gln767, Asp831, Lys721, and Phe832 residues of EGFR (Figure 3A); while Met769, Leu820, Leu694, Leu768, Pro770, Cys773, Gly772, Gly695, Asp831, Thr830, Glu738, Leu764, Met742, Lys721, Thr766, Ala719, Val702, and Gln767 residues of EGFR interacted with LTS0164690 (Figure 3B). Further, LTS0114552 interacted with Lys721, Met742, Val702, Leu820, Ala719, Met769, Leu768, Leu694, Pro770, Gly772, Thr830, Thr766, Leu764, and Asp831 residues of EGFR (Figure 3C). In addition, LTS0128805 bind with Lys721, Asp831, Leu764, Thr766, Met742, Thr830, Leu820, Ala719, Met769, Leu768, Gly772, Leu694, Pro770, and Val702 residues of EGFR (Figure 3D); while Lys721, Glu738, Gly833, Asp813, Asn818, Arg817, Gly772, Cys773, Tyr777, Asp776, Leu694, Ala719, Gln767, Leu768, Met769, Leu820, Val702, and Pro770 residues of EGFR interacted with LTS0058805 (Figure 3E). Further, LTS0007447 bind with Asp831, Lys721, Glu738, Leu764, Thr830, Met742, Thr766, Cys751, Gln767, Leu820, Ala719, Leu768, Met769, Gly772, Leu694, Val702, and Phe699 residues of EGFR (Figure 3F).

**Figure 4:**
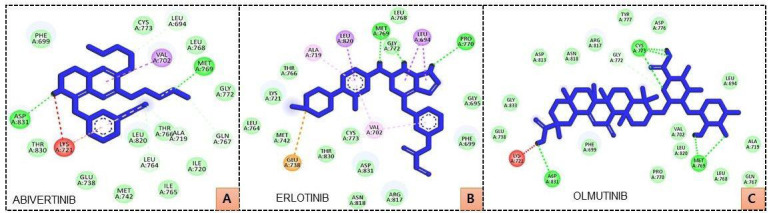
Interacting residues of EGFR with established inhibitors ABIVERTINIB (A), ERLOTINIB (B) and OLMUTINIB (C).

The interaction analysis of established inhibitors showed that Lys721, Glu738, Asp831, Gly833, Asp813, Asn818, Arg817, Gly772, Cys773, Tyr777, Asp776, Leu694, Pro770, Met769, Leu768, Gln767, Ala719, Leu820, Val702, and Phe699 residues are important in binding with EGFR (Figure 4A-C). Interestingly, the hit compounds LTS0058805, LTS0114552, LTS0128805, LTS0174203, LTS0007447, and LTS0164690 bind with these residues of EGFR (Figure 3A-F). Met769 was the common H-bonded residue of EGFR with LTS0058805, LTS0114552, LTS0128805, LTS0174203, LTS0007447, and LTS0164690 as well as the established inhibitors (Figure 3A-F and Figure 4A-C). Pro770, and Val702 residues of EGFR (Figure 3D); while Lys721, Glu738, Gly833, Asp813, Asn818, Arg817, Gly772, Cys773, Tyr777, Asp776, Leu694, Ala719, Gln767, Leu768, Met769, Leu820, Val702, and Pro770 residues of EGFR interacted with LTS0058805 (Figure 3E). Further, LTS0007447 bind with Asp831, Lys721, Glu738, Leu764, Thr830, Met742, Thr766, Cys751, Gln767, Leu820, Ala719, Leu768, Met769, Gly772, Leu694, Val702, and Phe699 residues of EGFR (Figure 3F).

Clinical medicine research is paying more attention to the utilization of natural ingredients and medicinal plants as cancer treatments [19]. One of the most researched herbal remedies, Glycyrrhiza (licorice) has various pharmacological effects [20]. It has shown that licorice-containing medicines are a safe, efficient, biocompatible, and cost-effective choice for the treatment of inflammatory illnesses and may even be used as adjunctive therapy in the early stages of primary malignancy [21,22]. Many studies have indicated that licorice flavonoid molecules have anti-cancer activity [22, 23,24]. In contrast to established EGFR inhibitors, which have been associated with numerous adverse effects, the hits documented in this study are licorice natural compounds and may exhibit a lower/no likelihood of inducing toxic effects.

## Conclusion:

The binding characteristics of selected G. glabra bioactive compounds (i.e., LTS0058805, LTS0114552, LTS0128805, LTS0174203, LTS0007447, and LTS0164690) concerning EGFR, have been thoroughly documented and merit further exploration for their potential application in cancer management.

## Figures and Tables

**Table 1:** Binding energy of know inhibitors with EGFR.

**EGFR inhibitors**	**Binding energy (Kcal/Mol)**
Abivertinib	-9.5
Olmutinib	-9.1
Icotinib	-9.1
Osimertinib mesylate	-8.6
Ravoxertinib	-8.5
Erlotinib	-8.5
Rociletinib	-8.4
Dacomitinib	-8.3
Lazertinib	-8.2
Canertinib	-7.9
Mobocertinib succinate	-7.8
Gefitinib	-7.7
Ulixertinib	-7.7
